# Constraint-induced movement therapy: trial sequential analysis applied to Cochrane collaboration systematic review results

**DOI:** 10.1186/1745-6215-15-512

**Published:** 2014-12-26

**Authors:** Greta Castellini, Silvia Gianola, Rita Banzi, Davide Corbetta, Roberto Gatti, Valeria Sirtori, Christian Gluud, Lorenzo Moja

**Affiliations:** Health Professional Science of Rehabilitation, University of Milan, Via Festa del Perdono, 7, 20122 Milan, Italy; Clinical Epidemiology Unit, IRCCS Orthopedic Institute Galeazzi, Via Riccardo Galeazzi, 4, 20161 Milan, Italy; IRCCS-Istituto di Ricerche Farmacologiche Mario Negri, Via La Masa, 19, 20156 Milan, Italy; Unit of Functional Recovery, San Raffaele Hospital, Via Olgettina 60, 20132 Milan, Italy; School of Physiotherapy, Vita-Salute San Raffaele University, Via Olgettina 58, 20132 Milan, Italy; Copenhagen Trial Unit, Centre for Clinical Intervention Research, Rigshospitalet, Copenhagen University Hospital, Blegdamsvej 9, DK-2100 Copenhagen, Denmark; Department of Biomedical Sciences for Health, University of Milan, Via Carlo Pascal, 36, 20133 Milano, Italy

**Keywords:** Stroke, Constraint-induced movement therapy, Meta-analysis, Random error, Trial sequential analysis

## Abstract

**Background:**

Trial sequential analysis (TSA) may establish when firm evidence about the efficacy of interventions is reached in a cumulative meta-analysis, combining a required information size with adjusted thresholds for conservative statistical significance. Our aim was to demonstrate TSA results on randomized controlled trials (RCTs) included in a Cochrane systematic review on the effectiveness of constraint-induced movement therapy (CIMT) for stroke patients.

**Methods:**

We extracted data on the functional independence measure (FIM) and the action research arm test (ARAT) from RCTs that compared CIMT versus other rehabilitative techniques. Mean differences (MD) were analyzed using a random-effects model. We calculated the information size and the cumulative Z-statistic, applying the O’Brien-Fleming monitoring boundaries.

**Results:**

We included data from 14 RCTs. In the conventional meta-analysis (seven trials, 233 patients), the effect of CIMT on FIM was reported as significant (MD 2.88, 95% CI 0.08 to 5.68; *P* = 0.04). The diversity-adjusted required information size was 142 patients, and the cumulative Z-score did not cross the trial sequential monitoring boundary for benefit (adjusted 95% CI -0.02 to 5.78). The effect of CIMT on ARAT (nine trials, 199 patients) was reported as significant (MD 7.78, 95% CI 1.19 to 14.37; *P* = 0.02). However, the diversity-adjusted required information size was 252 patients, and the Z-score did not cross the trial sequential monitoring boundary for benefit (adjusted 95% CI -0.06 to 15.62).

**Conclusions:**

Although conventional meta-analyses of CIMT reached statistical significance, their overall results remain inconclusive and might be spurious. Researchers should not be overconfident on CIMT efficacy based on the results of meta-analyses and derived recommendations.

## Background

Trial sequential analysis (TSA) is a methodology used to control for random errors in meta-analyses in order to critically appraise the effect of an intervention on a given outcome [1]. Conventional meta-analyses have a risk of false positive, neutral, or negative results that may lead to inaccurate conclusions regarding treatment effects [1–5]. Such spurious results may arise due to random errors generated by sparse data or repeated significance testing when updating meta-analyses with new trials [1, 5]. The latter risk can be assessed by several methods of testing (for example, when a multitude of interim analyses are conducted) in a single randomized controlled trial (RCT) [3, 5–7]. Spurious findings in meta-analyses can also result from systematic error (bias) due to deficiencies in study design and conduct such as inadequate randomization, lack of blinding, selective outcome reporting bias, and small study bias (which may be a proxy for the other bias mechanisms) [5, 8, 9]. Random errors and systematic errors can produce inaccurate and over-precise confidence intervals in meta-analyses, leading authors to overemphasize the real effectiveness of the investigated intervention. Although the scientific community has invested much efforts to expose the pitfalls of systematic errors (bias) in meta-analyses [5, 8, 9], it has largely ignored the issues of required information size and the risks of random errors in meta-analyses [1, 5, 10].

For each RCT, the number of events and patients required to make a reliable statistical inference must be estimated (the sample size guaranteeing sufficient power 1 – beta (the risk of type II error) to accept or rejects a certain intervention effect, which implies the clinically important target difference between the intervention groups and the standard deviation of the measurements, at a chosen risk of alpha (type I error)) [11]. Similarly, in order to control for the risk of random error, meta-analyses should include the calculation of a required information size, which is at least the sample size of an adequately powered single trial [10, 12, 13]. For continuous outcomes, the required information size of meta-analyses is based on: the minimal importance difference (MID) (*a priori* assumed intervention effect); standard error of the outcome; desired maximum risk of a false positive result (type I error, usually set as alpha = 0.05); and desired maximum risk of a false negative result (type II error, usually set as beta = 0.20 or 0.10) [1, 11]. The required information size of meta-analyses additionally depends on the amount of heterogeneity across the trials in the meta-analysis [14].

In systematic reviews, meta-analyses are commonly repeated to integrate accumulating data from newly published trials. Cochrane reviews, for example, require the regular updating of analyses to reflect current research. A statistically significant finding, however, is more likely in repeated analyses such that the actual risk of type I error in meta-analyses increases to between 10 and 30% [15–17]. Given this, retaining *P* values of less than 0.05 as evidence of ‘statistical significance’ can be highly misleading in meta-analyses that have not reached their required information size. A false positive result can be avoided by adjusting the threshold for statistical significance using conservative TSA monitoring boundaries, which are analogous to interim monitoring in a single RCT [1, 3, 13]. These monitoring boundaries are more conservative than conventional statistical boundaries, and adapt as new trials are published and meta-analyses are updated over time. TSA may quantify the reliability of the cumulative data in meta-analyses and provide important information on the number of additional patients required in future RCTs to reach ‘firm evidence’, a more reliable statistically significant results about the superiority, neutrality, or inferiority of the intervention. The total number of patients needed across included studies and future trials in order to reach this conclusion is called the required information size [1]. TSA shows the potential to be more reliable, supporting balanced interpretation of the overall effect size obtained by traditional meta-analysis techniques [10, 13].

The present study demonstrates TSA applied to a neurorehabilitation technique called constraint-induced movement therapy (CIMT). CIMT promotes the functional recovery of the upper arm in stroke patients through the forced use of the paretic limb; this is achieved by restraining the sound limb with a glove and special armrest [18–20]. Our updated Cochrane review with conventional meta-analyses reported a statistically significant effect of CIMT on arm motor function as measured through the Action Research Arm Test (ARAT), and a non-statistically significant effect on disability using the Functional Independence Measure (FIM) [4]. The majority of the trials, however, featured major risks of bias. Thus, the statistically significant findings may be attributed to systematic error (bias) and random error (play of chance), or represent a true treatment effect.

In our present study, we considered these risks of bias in the calculation of the required information size, but disregarded them when applying TSA to the RCTs comparing CIMT with traditional rehabilitation in stroke patients [21]. We critically appraised the results of our Cochrane review to determine whether firm evidence could be reached regarding the efficacy of CIMT on disability and arm motor function. If the data were inconclusive, we calculated the number of patients needed in future RCTs to attain the required information size or reach the area of futility. We aimed to illustrate the importance of accounting for the risk of random error in cumulative meta-analyses, and to provide a tool for quantifying the amount of additional evidence required to reach firm evidence. Moreover, we sought to demonstrate to authors of meta-analyses that the incorporation of TSA can lead to more conservative results, thereby avoiding the premature conclusion of treatment efficacy or non-efficacy.

## Methods

This manuscript is based on an update of a Cochrane systematic review published in The Cochrane Library [4]. Briefly, we analyzed RCTs or quasi-randomized studies that compared CIMT to other rehabilitative techniques (physical therapy or occupational therapy). To be included in the review, participants had to be older than 18 years of age with a diagnosis of ischemic or hemorrhagic stroke and upper limb paresis with sustained ability to move the affected hand. The search strategy is available in the Appendix. We considered all types of CIMT, irrespective of the number of hours of training per day, number of hours of constraint per day, duration of treatment, and type of exercise used in the training section. Two independent reviewers (GC and DC) extracted and analyzed the data. A third review (LM) author resolved any disagreements.

In the updated review, for analyses on disability, we focused on data using the FIM [22]; for arm motor function, we extracted data measured by the ARAT (the original review did not specify FIM and ARAT in the inclusion criteria thus the updated review excluded all trials in the original review using different outcomes measure scales.) [23]. We decided to select these outcomes because the aim of CIMT is to improve functional recovery and potential motor ability. Upper extremities are important to most daily activities, making it difficult to select a measure that considers all types of activities. FIM refers to a measurement of global disability; it can be used to represent functional activities involving the upper extremities (such as eating, bathing, and dressing) of a patient. Because some trials might have adopted modified versions of FIM and ARAT with a different number of items, we estimated the FIM score for each study (scores presented in the studies were divided by the total score of the subscales or items used). To better evaluate upper extremities, we assessed the quality of movement and functionality with ARAT, which was reported in most RCTs included in the original review and its update [4, 21].

### Risk of bias assessment

We used all the Cochrane Handbook domains for assessing the risk of bias [24]. We sub-grouped trials according to the level of risk of bias based on the most recent risk of bias assessment in the updated review according to the Cochrane Handbook of Systematic Reviews of Interventions [5]. Two independent reviewers (GC and DC) assessed the risk of bias; disagreements were resolved by consulting a third author (LM). We decided to rate RCTs as at a lower risk of bias when the domains sequence generation, concealment of allocation, and blinding were adequate, even if other domains were with high risk of bias. An RCT was evaluated as having a high risk of bias if one or more of these domains were judged to be at a high risk of bias, or when the reporting was unclear.

### Statistical methods

#### Meta-analysis

Data were summarized as a mean difference (MD) with 95% confidence intervals (CI) using Review Manager (RevMan version 5.0.25 The Cochrane Collaboration, Copenhagen:The Nordic Cochrane Centre, Denmark, 2014.) [25]. The inconsistency (I^2^) statistic and the diversity (D^2^) statistic were used to assess the degree of heterogeneity among trials in each analysis [5, 14]. Data were summarized in a meta-analysis using the random-effects models described by DerSimonian and Laird [26], irrespective of I^2^ values, assuming clinical heterogeneity to be compelling in this clinical setting due to differences in the types of patient populations (for example, stroke severity), interventions (duration and intensity), and controls (for example, differences in standard physiotherapies).

#### Trial sequential analysis

For each meta-analysis, we calculated the diversity-adjusted required information size and applied trial sequential monitoring boundaries with TSA version 0.9 beta (Copenhagen Trial Unit, Centre for Clinical Intervention Research, Copenhagen, Denmark. 2011) [13].The information size was based on an alpha of 0.05, a beta of 0.20 (power of 80%), the variance of the intervention effect between studies, and the MID. The methods used to determine the MID are presented in the ‘Methods to estimate the MID in the TSA’ section. We set the conservative trial monitoring boundaries by Lan-DeMets-O’Brien-Fleming as the α-spending function [1, 27]. We calculated the cumulative Z-curve of each cumulative meta-analysis and plotted it against the above monitoring boundaries according to the random-effects models. In general, one of the following TSA results may occur. The crossing of the cumulative Z-curve (the series of Z-statistics after each consecutive trial) into the trial sequential monitoring boundary for benefit indicates that a sufficient level of evidence has been reached, and no further trials may be needed to demonstrate the superiority of the intervention. If the cumulative Z-curve does not cross any of the trial sequential monitoring boundaries, however, there is likely insufficient evidence to reach a conclusion, and additional trials may be required [10, 28]. Here, the question concerns whether the Z-curve has reached the futility area. If not, the TSA can provide additional information on the size (number of patients) of future RCTs to reach a definitive conclusion. In our study, we compared Z-scores obtained from TSA and RevMan to check for potential inconsistencies.

### Methods to estimate the minimal important difference in the trial sequential analysis

MID has been defined as ‘the smallest difference in score in the domain of interest that patients perceive as important, either beneficial or harmful, and which would lead the clinician to consider a change in the patient’s management’ [29]. The magnitude of the MID is linked to the intervention effect size, and their relationship determines the clinical relevance of an intervention. There are two methods to establish the MID. The first and preferred method, the ‘anchor-based approach’, is used when the MID is derived from a study that is evaluated as interpretable, valid, and generalizable [30]. In this case, a low-cost intervention without adverse effects would likely be used even if the MID was low. Alternatively, an expensive intervention with potential adverse events would only be of interest if the MID was large. The second method, the ‘distribution-based approach’ [31, 32], may assume that a change equal to 0.5 of the standard deviation on the instrument score is representative of the ‘true’ MID. An effect size of 0.5 of the standard deviation is usually described as a moderate effect size [33].The MID can be estimated by multiplying the effect size of 0.5 by the pooled standard deviation between groups. The formulas to calculate the effect size and the pooled standard deviation are provided below [34]:
1

Where ES is effect size, X_G1_ is mean group 1, X_G2_ is mean group 2, and S_pooled_ is pooled standard deviation.

To calculate the pooled standard deviation, the following formula is used:
2

Where S_1_ is standard deviation group 1, S_2_ is standard deviation group 2, n_1_ is the sample size for group 1, n_2_ is the sample size for group 2.
3

Preferably, the reference standard MID should be established by the anchor-based method rather than the distribution-based method. The anchor-based method reflects the point of view of the patients [35, 36], while the distribution-based method may not. If an RCT presented an anchor-based MID that was positively evaluated by the authors, we used this value. Otherwise, we implemented the distribution-based method.

## Results

**Figure 1:**
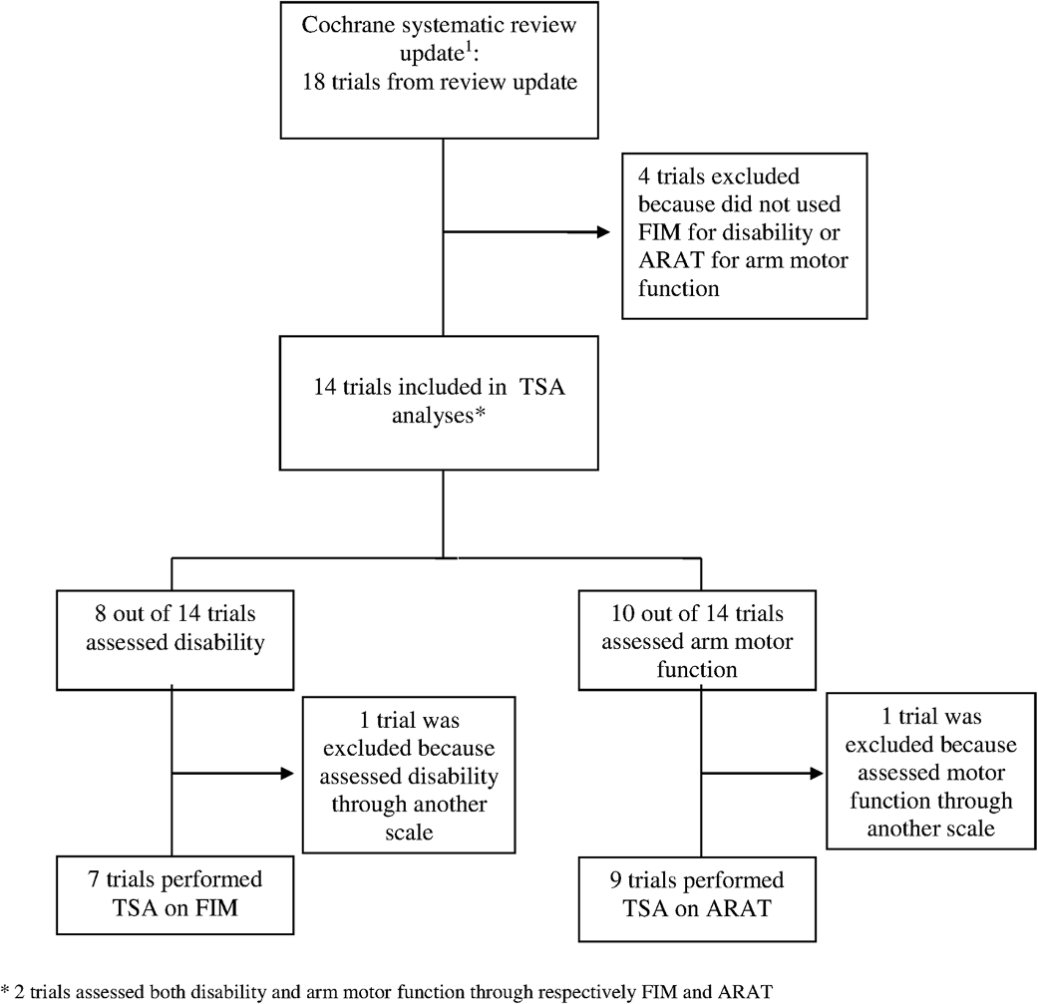
**Flow diagram of trials presenting selection for inclusion.** Of the 14 trials included in the trial sequential analyses, two assessed both disability and arm motor function through FIM and ARAT, respectively. Abbreviations: FIM, Functional Independence Measure; ARAT, Action Research Arm Test; TSA, Trial Sequential Analysis. 1. Corbetta D, Sirtori V, Moja L, Gatti R: Constraint-induced movement therapy in stroke patients: systematic review and meta-analysis. *Eur J Phys Rehabil Med* 2010.

We excluded four [37–40] of the 18 trials included in the updated Cochrane Systematic Review [4] as they did not report on the two selected outcomes (FIM and ARAT). Accordingly, we included 14 trials in our present study reporting either FIM or ARAT. Figure 1 shows the study flow. The characteristics of the trials are available in the original and updated reviews [4, 21].

### Risk of bias assessment

All the included trials were at high risk of bias (Table 1). Four trials were not adequately randomized [41–44] and only three were adequately concealing the allocation [45–47]. A total of 13 trials claimed to have blinded the outcome assessment, however, the blinding may have been compromised by inadequate randomization. Three trials were at lower risk of bias due to adequate randomization and blinding [45–47].

**Table 1:** **Risk of bias assessment of included randomized and quasi-randomized controlled trials**

Trial	Sequence generation	Allocation concealment	Blinding of assessors	Lower or high risk of bias
Atteya *et al*. [41]	Inadequate	Unclear	Adequate	High^b^
Dahl *et al*. [48]	Adequate	Unclear	Adequate	High
Dromerick *et al*. [52]	Adequate	Unclear	Adequate	High
Dromerick *et al*. [42]	Inadequate	Unclear	Adequate	High
Hammer and Lindmark [53]	Adequate	Unclear	Inadequate	High
Lin *et al*. [47]	Adequate	Adequate	Adequate	Lower^a^
Lin *et al*. [46]	Adequate	Adequate	Adequate	Lower
Myint *et al*. [45]	Adequate	Adequate	Adequate	Lower
Page *et al*. [43]	Inadequate	Unclear	Adequate	High
Page *et al*. [55]	Adequate	Unclear	Adequate	High
Page *et al*. [54]	Adequate	Unclear	Adequate	High
Ploughman and Corbett [49]	Adequate	Unclear	Adequate	High
Wu *et al*. [44]	Inadequate	Unclear	Adequate	High
Wu *et al*. [50]	Adequate	Unclear	Adequate	High

^a^Lower risk of bias when the domains sequence generation, concealment of allocation, and blinding of assessors were adequate.

^b^High risk of bias when one or more of these domains were judged as inadequate or the reporting was unclear.

### Disability - Functional Independence Measure

**Figure 2:**
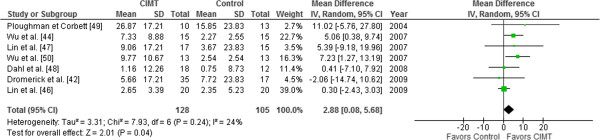
**Meta-analysis of constraint-induced movement therapy (CIMT) versus control on disability (FIM).** Effect of CIMT compared with usual care on disability, assessed using Functional Independence Measure (FIM) in adult stroke patients. The meta-analysis included 233 stroke patients. The black diamond does not cross the vertical line and showed an effect size of 2.88 (95% confidence interval 0.08 to 5.68), which is only significant (*P* = 0.04) using conventional meta-analytic methods. Heterogeneity expressed as I^2^ was 24%. Abbreviations: CIMT, Constraint induced movement therapy; FIM, Functional independence measure.

Eight out of 14 trials were included in our study assessed disability; seven trials [42, 44, 46–50], comprising 233 patients with stroke, assessed disability through FIM or its modified version, and one RCT [45] assessed disability through another scale, the Barthel Index, and was excluded. Meta-analysis of CIMT versus traditional rehabilitation showed a statistically significant benefit of CIMT on FIM (random-effects model, MD 2.88 points, 95% CI 0.08 to 5.68; I^2^ = 24%) (Figure 2).

**Figure 3:**
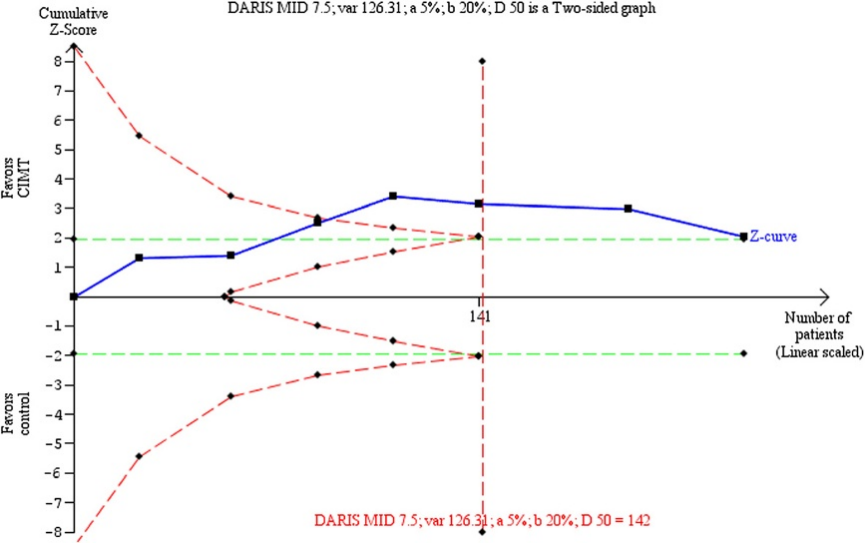
**Trial sequential analysis of constraint-induced movement therapy (CIMT) versus control for disability (FIM).** Diversity-adjusted required information size of 142 patients calculated on basis of 7.50 MID of FIM (see text); variance of 126.31; alpha = 5%; beta = 20%, and diversity (D or rather D^2^) of 50%. The blue cumulative Z-curve crosses the inward sloping red trial sequential monitoring boundary for benefit and the required information size. The horizontal green lines illustrate the conventional level of statistical significance (two-sided alpha = 0.05), which was intersected after the third trial. Although with 199 patients randomized we have sufficient evidence to accept an effect of CIMT on FIM, after the seventh trial, the cumulative Z-score comes back toward the null effect, just above the trial sequential monitoring boundaries score. The analysis and the figure were performed with TSA software. Legend. Square symbol: z-score for single study; Diamond symbol: trial sequential monitoring boundary for benefit score for single study. Abbreviations: DARIS, diversity-adjusted required information size; MID, minimal important difference; var, variance; D, diversity; a, alpha; b, beta.

Although one study had an established MID based on the anchor-based approach [51], its population did not follow our inclusion criteria: stroke patients included in our review had a higher initial FIM score, which was necessary to be able to perform the CIMT technique. We found one study in the literature that estimated an MID for stroke patients of 22 points on the total FIM (total is 126 points) [51]. However, such improvement is not plausible in patients with a high initial FIM score; in other words, the higher the FIM score, the least likely that the MD is clinically evident. Therefore, a distribution-based approach was performed to determine the MID. We selected the study by Lin *et al*. [47] as the best trial candidate to derive the MID; it had a lower risk of bias and acceptable external validity. The estimated MID was 7.85 points. If we do not consider the risks of bias, the diversity-adjusted required information size calculated was 142 patients (Figure 3). If the risks of bias are disregarded, the TSA supported the acceptance of CIMT as an effective intervention to improve FIM; with the publication of the sixth trial, the cumulative Z-curve crossed the information size and the trial sequential monitoring boundary for benefit. However, the subsequently published RCTs had less extreme results compared to the first studies and moved the cumulative Z-curve back toward the no-difference line, raising doubts about the conclusiveness of the information size on the potential benefit of CIMT on FIM. The final cumulative Z-score did not cross the trial sequential monitoring boundary for benefit (adjusted 95% CI -0.02 to 5.78).

### Arm motor function - Action Research Arm Test

**Figure 4:**
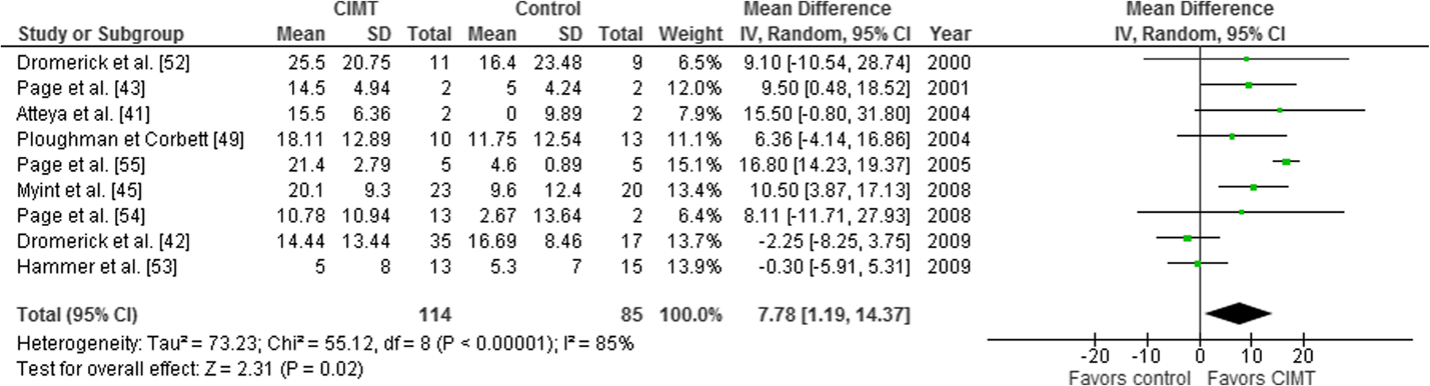
**Meta-analysis constraint-induced movement therapy (CIMT) versus control on arm motor function (ARAT).** Effect of CIMT compared with usual care on arm motor function, assessed using Action Research Arm Test (ARAT) in adult stroke patients. The meta-analysis included 199 stroke patients. The black diamond does not cross the vertical line and showed an effect size of 7.78 (95% CI 1.19 to 14.37), which is significant (*P* = 0.02) using conventional meta-analytic methods. Heterogeneity expressed as I^2^ 85% was high. Abbreviations: CIMT, Constraint induced movement therapy; ARAT, action research arm test.

Nine trials [41–43, 45, 49, 52–55], totaling 199 patients, assessed arm motor function using ARAT; five RCTs were excluded because one [48] assessed motor function through the Wolf Motor Function Test (WMFT) and four trials did not assess arm motor function [44, 46, 47, 50]. Meta-analyses showed a statistically significant CIMT effect on arm motor function (random effects, MD 7.78 points, 95% CI 1.19 to 14.37; I^2^ = 85%) (Figure 4).

**Figure 5:**
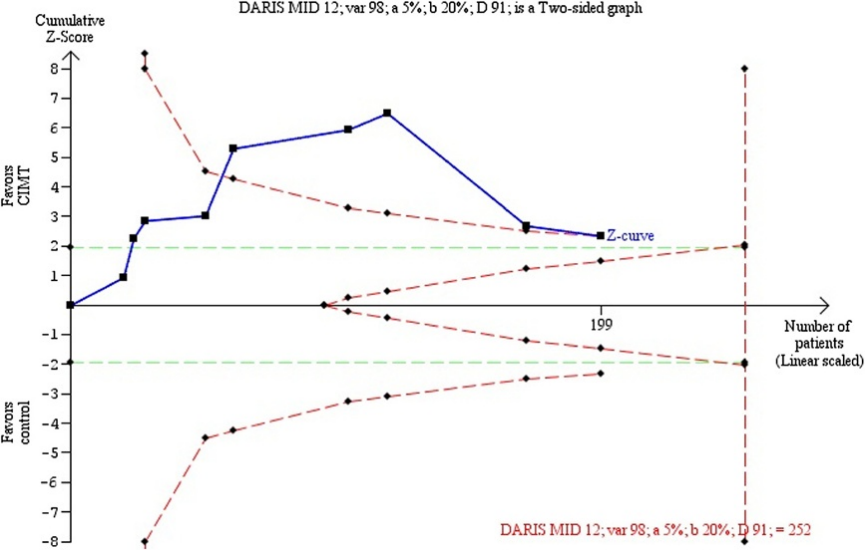
**Trial sequential analysis constraint-induced movement therapy (CIMT) versus control for arm motor function (ARAT).** Diversity-adjusted required information size of 252 patients calculated on basis of 12 MID of ARAT; a variance of 98; alpha = 5%; beta = 20%, and a diversity (D or rather D^2^ of 91%). The blue cumulative Z-curve crosses first the horizontal green line of conventional statistical significance (alpha = 0.05) during the second trial and then crossed the inward sloping red trial sequential monitoring boundary for benefit during the fifth trial. After the ninth trial, the cumulative Z-score for 199 patients of 2.31 is just below the trial sequential monitoring boundary score of 2.33. With 199 out of 252 (79.0%) patients randomized we still have insufficient evidence to accept or reject an effect of CIMT on ARAT, although it looked promising during the fifth, sixth, and seventh trials (disregarding the risks of bias). The analysis and the figure were made with the TSA software. Legend. Square symbol: z-score for single study; Diamond symbol: trial sequential monitoring boundary for benefit score for single study. Abbreviations: DARIS, diversity-adjusted required information size; MID, minimal important difference; var, variance; D, diversity; a, alpha; b, beta.

An anchor-based approach was used to determine the MID in the RCT by Lang *et al*. [56]. The estimated MID was 12 points, corresponding to the diversity-adjusted required information size of 252 patients (Figure 5). If the risks of bias are disregarded, the cumulative Z-curve crossed the trial sequential monitoring boundaries after the fifth trial, demonstrating a significant intervention effect. However, the final cumulative Z-score did not cross the trial sequential monitoring boundary for benefit (adjusted 95% CI -0.06 to 15.62).

Although CIMT looked promising during the fifth, sixth, and seventh trials, the intervention no longer showed a statistically significantly effect on ARAT in the eighth and ninth published trials. Additional patients (increased from 199 to 252), might provide more evidence to either accept or reject an effect of CIMT on ARAT, decreasing doubts about CIMT consistency.

## Discussion

The implications of our Cochrane review on the effectiveness of CIMT for practice were limited by numerous underpowered studies with a small sample size. Furthermore, the results could have been influenced by systematic error in studies with a high risk of bias as well as random errors [1]. Our conventional meta-analysis of the primary outcome, disability, in the updated review showed no statistically significant effect of CIMT on FIM, in contrast with our observation in the previous version of the review. A meta-analysis on a secondary outcome, arm motor function, showed a moderate statistically significant effect on ARAT [4]. We felt that our conclusions were highly dependent on the last small published RCT, which led to the appearance of spuriously favorable results in our primary meta-analysis. In the present study, meta-analyses and TSAs were used to better explore the evidence on the effects of CIMT on disability and arm motor function in patients with stroke. Although the results of the meta-analysis on the primary outcome were statistically significant, the clinical relevance was questionable due to the small effect size. In fact, for the disability outcome, the TSA data suggested that the information size was sufficient. The cumulative Z-score, however, moved towards null effect, while a few additional patients seemed necessary to reach firm conclusion on the arm motor function outcome. More evidence on treatment efficacy is required to decrease the socioeconomic burden of this pathological condition. We chose to explore disability and arm motor function as the main outcomes due to their importance not only for patients and their quality of life, but also for clinicians, families, healthcare systems, and society. An effective treatment that decreases disability level and increases arm motor function can improve patient ability in daily life, their life satisfaction, and quality of life, and reduce family load, social assistance, hospitalization, and economic social charge.

Two important caveats to these results arises from concerns about the state of science of RCTs that explored the efficacy of CIMT. The positive finding of CIMT may have been enhanced by the high percentage of low quality and small studies. Reporting and publication biases, particularly the bias towards publishing studies with positive findings, may have further affected our results. In fact, our study shows that more recent publications had less extreme results compared to earlier studies, such that the latter may present findings that are out of proportion to the truth [57]. Highly beneficial results are most tantalizing and attractive to investigators and editors. Replication studies with intermediate, potentially less extreme results may then be published, filling in the gap between the early impressive and the later modest effects.

Systematic reviews and meta-analyses are considered to be the best available evidence to inform clinical decision-making, although the best available evidence may not be synonymous with ‘sufficient evidence’ or ‘strong evidence’ [12, 28]. The interpretation of meta-analyses should not be simplistic. About 25% of conventional meta-analyses, which include a small number of events and patients, may falsely report the estimated intervention effects as statistically significant. Empirical evidence also shows that large pooled intervention effects observed in early positive meta-analyses tend to dissipate as more evidence is accumulated [28]. When a meta-analysis includes a small number of trials and a small number of patients, random errors can cause spurious findings [10]; paucity of studies and patients is common in the rehabilitation field [58]. To the best of our knowledge, this study is the first attempt to apply TSA to the neurorehabilitation field, and demonstrates TSA to be a useful tool to adjust for random-error risk. Authors, peer reviewers, and readers should carefully consider the statistically significant results proposed by meta-analyses conducted on the early phase of research on innovative therapies [59].

TSA does present limits. Only trials with the same outcome measure can be included, thereby excluding a part of the available information: further TSA techniques should be developed in order to cumulate standardized MDs, an effect size which overcomes differences across measures. Our findings should be considered with caution, since they are driven by assumptions on the MID and can only be generalized to similar patient populations. An important issue is the way in which the MID was established. We used a distribution-based method when the literature did not provide a clear MID. However, this methodology may not be optimal.

TSA focuses on random error, although bias is also an important variable that must be considered when recommending an intervention. Most of the included trials featured a high risk of bias; three were considered to have a lower risk of bias. None of the trials were judged to be at a low risk of bias for all seven domains [24]. Most of the included RCTs (11 out of 14 included studies) did not report allocation concealment. Although the allocation concealment is known to be an important component of trial design, Foley *et al*. [60] underline that most trials in stroke rehabilitation that were published after the release of the CONSORT statement did not provide any description of the concealment of the allocation sequence. Results could be more conservative if the trials were conducted with a high methodological quality, taking into account all types of bias, which are not all reported here. Well-designed trials with a low risk of bias in all seven bias domains [24] may help conduct more conservative TSAs and produce more reliable results for quantifying the amount of additional evidence.

Moreover, we extracted data from trials in a previously published systematic review, without considering the potential availability of new studies since its publication. We will add these trials to the next update of the meta-analysis where we intend to conduct TSA on the outcomes and a Grading of Recommendations Assessment, Development and Evaluation (GRADE) analysis for the quality of evidence. The inclusion of additional trials may show conclusive evidence, suggesting that no further trials are needed if futility is reached, or that superiority is proven. Before conducting new trials, investigators should collect available evidence on past trials and design their trial against the results of TSA. Recently, some trials [61, 62] have randomized patients to different types of CIMTs. This assumes that CIMT is effective for patients with stroke, although there remains insufficient evidence to firmly establish the intervention’s potential benefits. Exposing patients to a therapy in its infancy exposes them to risks as the therapy can be ineffective or detrimental. If new trials are published, it is easy to re-estimate the additional number of patients required to obtain firm evidence in the meta-analysis, thereby guiding trialists about the optimal sample size to adopt [63, 64]. In some cases, TSA may stop the implementation of trials accumulating redundant evidence [65].

## Conclusions

Evidence on the effects of CIMT reported in our Cochrane review [4] may be optimistic since the replication studies conducted in the last years did not confirm any statistically significant beneficial effects of CIMT. Publication bias and the appearance of spuriously favorable results in the early phase of the accumulation of scientific evidence are well-established problems in biomedical research [57, 66]. High-quality RCTs are needed to resolve uncertainties surrounding the effectiveness of CIMT. The risk of random error in meta-analyses is becoming increasingly important as some authors advocate moving towards smaller RCTs with relaxed type I errors. TSA can detect random error in the existing cumulative meta-analysis and identify the need for future trials. We recommend the use of TSA along with systematic error assessment tools.

## Appendix

### MEDLINE (Ovid) search strategy

The following search strategy, which was developed by the Cochrane Stroke Group Trials Search Coordinator, was used for the Medical Literature Analysis and Retrieval System Online database (MEDLINE) (Ovid), and was adapted for the Cochrane Central Register of Controlled Trials (CENTRAL):

1. exp cerebrovascular disorders/ or brain injuries/ or brain injury, chronic/
2. (stroke$ or cva or poststroke or post-stroke).tw.
3. (cerebrovasc$ or cerebral vascular).tw.
4. (cerebral or cerebellar or brain$ or vertebrobasilar).tw.
5. (infarct$ or isch?emi$ or thrombo$ or emboli$ or apoplexy).tw.
6. 4 and 5
7. (cerebral or brain or subarachnoid).tw.
8. (haemorrhage or hemorrhage or haematoma or hematoma or bleed$).tw.
9. 7 and 8
10. hemiplegia/ or exp paresis/
11. (hempar$ or hemipleg$ or paresis or paretic or brain injur$).tw.
12. 1 or 2 or 3 or 6 or 9 or 10 or 11
13. exp upper extremity/
14. (upper limb$ or upper extremit$ or arm or shoulder or hand or axilla or elbow$ or forearm$ or finger$ or wrist$).tw.
15. 13 or 14
16. restraint, physical/
17. exercise movement techniques/ or exercise/ or exercise therapy/
18. immobilization/
19. physical therapy techniques/
20. (constrain$ or restrain$ or immobili$).tw.
21. (mCIMT or CIT or “CI therapy” or “forced use”).tw.
22. recovery of function/
23. splints/ or casts, surgical/
24. or 17 or 18 or 19 or 20 or 21 or 22 or 23
25. 12 and 15 and 24

### EMBASE (Ovid) search strategy

The following search strategy was used for the Excerpta Medica dataBASE (EMBASE) (Ovid):

1. cerebrovascular disease/ or basal ganglion hemorrhage/ or cerebral artery disease/ or cerebrovascular accident/ or stroke/ or exp carotid artery disease/ or exp brain hematoma/ or exp brain hemorrhage/ or exp brain infarction/ or exp brain ischemia/ or exp intracranial aneurysm/ or exp occlusive cerebrovascular disease/ or exp brain injury/
2. (stroke$ or cva or poststroke or post-stroke).tw.
3. (cerebrovasc$ or cerebral vasc$).tw.
4. (cerebral or cerebellar or brain$ or vertebrobasilar).tw.
5. (infarct$ or isch?emi$ or thrombo$ or emboli$ or apoplexy).tw.
6. 4 and 5
7. (cerebral or brain or subarachnoid).tw.
8. (haemorrhage or hemorrhage or haematoma or hematoma or bleed$).tw.
9. 7 and 8
10. hemiplegia/ or hemiparesis/ or paresis/
11. (hemipleg$ or hemipar$ or paresis or paretic or brain injur$).tw.
12. 1 or 2 or 3 or 6 or 9 or 10 or 11
13. exp arm/
14. (upper limb$ or upper extremit$ or arm or shoulder or hand or axilla or elbow$ or forearm$ or finger$ or wrist$).tw.
15. 13 or 14
16. exp exercise/ or exp kinesiotherapy/ or physiotherapy/ or immobilization/
17. (restrain$ or constrain$ or immobili$).tw.
18. (mCIMT or CIT or CI therapy or “forced use”).tw.
19. dynamic splint/ or plaster cast/ or splint/
20. (splint$ or cast or casts).tw.
21. or/16-20
22. 12 and 15 and 21

### CINAHL search strategy

The following search strategy was used for the Cumulative Index to Nursing and Allied Health Literature database (CINAHL):

1. exp cerebrovascular disorders/
2. stroke$.tw.
3. cva$.tw.
4. cerebrovasc$.tw.
5. cerebral vascular$.tw.
6. (cerebral or cerebellar or brain$ or vertebrobasilar).tw.
7. (infarct$ or isch?emi$ or thrombo$ or emboli$ or vasospasm$ or apople$).tw.
8. 6 and 7
9. (cerebral or intracerebral or intracranial or parenchymal or brain$ or intraventricular or periventricular or cerebellar or infratentorial or supratentorial or subarachnoid).tw.
10. (Haemorrhage or hemorrhage or haematoma or hematoma or bleed$ or aneurysm).tw.
11. 9 and 10
12. trans$ isch?emic attack$.tw.
13. tia.tw.
14. hemiplegia/
15. brain injuries/ or left hemisphere injuries/ or right hemisphere injuries/
16. (hemipar$ or hemipleg$ or paresis or paretic or brain injur$).tw.
17. 1 or 2 or 3 or 4 or 5 or 8 or 11 or 12 or 13 or 14 or 15 or 16
18. exp arm/
19. (upper limb$ or upper extremit$ or arm or shoulder or hand or axilla or elbow$ or forearm$ or finger$ or wrist$).tw.
20. 18 or 19
21. Restraint, Physical/
22. Immobilization/
23. “Taping and Strapping”/
24. exp Exercise/
25. exp Therapeutic Exercise/
26. Physical Therapy/mt [Methods]
27. splints/ or slings/ or casts/
28. “Task Performance and Analysis”/
29. (constrain$ or restrain$ or immobil$).tw.
30. (mCIMT or CIT or “CI therapy” or “forced use”).tw.
31. 21 or 22 or 23 or 24 or 25 or 26 or 27 or 28 or 29 or 30
32. 17 and 20 and 31

### PEDro search strategy

The following search strategy was used for the Physiotherapy Evidence Database (PEDro):

PEDro is a web-based database of randomized controlled trials and systematic reviews relevant to physiotherapy. The following search strategy was used.

Abstract and Title: constraint, stroke, cva, poststroke, hemi, brain injur, *matoma, bleed, cerebrovasc, cerebral, brain, infarct, thrombo.

Body part: upper arm, shoulder or shoulder girdle/forearm or elbow/hand or wrist.

All search terms in the title or abstract were combined with body part descriptors using the AND operator.

## Abbreviations

ARAT: Action research arm test
CI: confidence interval
CIMT: Constraint-induced movement therapy
DARIS: diversity-adjusted required information size
FIM: Functional Independence Measure
GRADE: Grading of Recommendations Assessment, Development and Evaluation
MD: mean difference
MID: Minimal important difference
RCT: Randomized controlled trial
SD: standard deviation
TSA: Trial sequential analysis
Var: variance.
